# Correlation Network Analysis Reveals Relationships between MicroRNAs, Transcription Factor* T-bet*, and Deregulated Cytokine/Chemokine-Receptor Network in Pulmonary Sarcoidosis

**DOI:** 10.1155/2015/121378

**Published:** 2015-11-30

**Authors:** Tereza Dyskova, Regina Fillerova, Tomas Novosad, Milos Kudelka, Monika Zurkova, Petr Gajdos, Vitezslav Kolek, Eva Kriegova

**Affiliations:** ^1^Department of Immunology, Faculty of Medicine and Dentistry, Palacký University, 77515 Olomouc, Czech Republic; ^2^IT4Innovations National Supercomputing Center and Faculty of Electrical Engineering and Computer Science, Department of Computer Science, VŠB-Technical University of Ostrava, 70800 Ostrava, Czech Republic; ^3^Department of Respiratory Diseases, Faculty of Medicine and Dentistry, Palacký University and Faculty Hospital, 77900 Olomouc, Czech Republic

## Abstract

Sarcoidosis is an inflammatory granulomatous disease with unknown etiology driven by cytokines and chemokines. There is limited information regarding the regulation of cytokine/chemokine-receptor network in bronchoalveolar lavage (BAL) cells in pulmonary sarcoidosis, suggesting contribution of miRNAs and transcription factors. We therefore investigated gene expression of 25 inflammation-related miRNAs, 27 cytokines/chemokines/receptors, and a Th1-transcription factor* T-bet* in unseparated BAL cells obtained from 48 sarcoidosis patients and 14 control subjects using quantitative RT-PCR. We then examined both miRNA-mRNA expressions to enrich relevant relationships. This first study on miRNAs in sarcoid BAL cells detected deregulation of* miR-146a*,* miR-150*,* miR-202*,* miR-204*, and* miR-222* expression comparing to controls. Subanalysis revealed higher number of* miR-155*,* let-7c* transcripts in progressing (*n* = 20) comparing to regressing (*n* = 28) disease as assessed by 2-year follow-up. Correlation network analysis revealed relationships between microRNAs, transcription factor* T-bet*, and deregulated cytokine/chemokine-receptor network in sarcoid BAL cells. Furthermore,* T-bet* showed more pronounced regulatory capability to sarcoidosis-associated cytokines/chemokines/receptors than miRNAs, which may function rather as “fine-tuners” of cytokine/chemokine expression. Our correlation network study implies contribution of both microRNAs and Th1-transcription factor* T-bet* to the regulation of cytokine/chemokine-receptor network in BAL cells in sarcoidosis. Functional studies are needed to confirm biological relevance of the obtained relationships.

## 1. Introduction

Pulmonary sarcoidosis is an inflammatory disorder of unknown etiology characterized by the accumulation of activated Th1/Th17 lymphocytes and macrophages in the alveoli and subsequent granuloma formation [1–3]. The key role in the pathogenesis of sarcoidosis is played by proinflammatory cytokines and chemokines, molecules crucially involved in the activation of immune and inflammatory cells and their trafficking to the site of disease [4]. However, there is still limited information about the regulation of the cytokine/chemokine-receptor network in pulmonary sarcoidosis and its phenotypes.

There is a growing body of evidence that the regulation of inflammatory response is a very complex process involving coordinated participation of multiple regulation systems, such as an integrated network of microRNAs (miRNAs) and transcription factors [5, 6]. The emerging role of miRNAs, a class of single-stranded noncoding RNAs of 19–25 nucleotides in length, in regulation of inflammatory response has been already reported in chronic pulmonary diseases such as asthma [7] and chronic obstructive pulmonary disease [8]. In sarcoidosis, altered miRNA pattern has been reported in lung tissues [9], peripheral blood mononuclear cells [9–11], and serum [10]. However, there is no information regarding the miRNA pattern in bronchoalveolar lavage (BAL) cells and their regulatory capability related to cytokine/chemokine-receptor network in pulmonary sarcoidosis. Also, a Th1-transcription factor* T-bet* has emerged as key regulator of crucial immune genes such as interferon- (IFN-) *γ* and chemokine receptor CXCR3 in sarcoid inflammation [12–14] as well as in other inflammatory conditions [15–17]. However, no information about the possible cooperation of this Th1-transcription factor and inflammation-related microRNAs in regulation of cytokine/chemokine-receptor network in BAL cells in sarcoidosis exists yet.

In this study, we aimed to investigate the gene expression pattern of candidate inflammation-related miRNAs in BAL cells obtained from sarcoidosis patients and control subjects. In order to assess the possible contributions of miRNAs as posttranscriptional regulators and* T-bet* as a driver Th1-transcription factor on sarcoid inflammation, we searched for relationships between miRNAs and* T-bet* with sarcoidosis-associated cytokine/chemokine-receptor network in BAL cells obtained from patients with sarcoidosis and subgroups with progressing and regressing disease as assessed by 2-year follow-up. We believe that understanding the transcriptional and posttranscriptional regulation of cytokine/chemokine-receptor network could shed light on the cause and progression of pulmonary sarcoidosis and other inflammatory and autoimmune diseases and eventually lay the groundwork for therapeutic options.

## 2. Materials and Methods

### 2.1. Subjects

Patients were further subdivided according to the disease development as assessed by 2-year follow-up. BAL was performed according to a standard protocol [18] in 48 patients with pulmonary sarcoidosis (S) and 14 control subjects (C) of Czech origin. The diagnosis of sarcoidosis was determined in compliance with the criteria from the Statement on Sarcoidosis [19]. No patient received corticosteroid treatment before BAL. Patients were further subdivided according to the disease development as assessed by the 2-year follow-up: (i) patients with progressing disease (Prog, *n* = 20) and (ii) those where the regression was achieved (Reg, *n* = 28). The control group consisted of 14 subjects undergoing BAL as a part of clinical investigation for psychogenic cough, cough associated with gastroesophageal reflux disease and lung hypertension. For clinical and laboratory characteristics of enrolled patients and control subjects, see Table 1.

All patients gave their informed consent for the use of BAL, taken primarily for diagnostic evaluation, for the purpose of this study. The local ethical committee of Palacký University and Faculty Hospital, Olomouc, approved the study.

### 2.2. BAL Sample Processing, miRNA/mRNA Isolation, and Reverse Transcription

BAL cells were separated from the fluid by centrifugation and total RNA was isolated from unseparated BAL cells with mirVana miRNA kit (Ambion, Austin, USA); RNA quality and quantity were measured by 2100 Bioanalyzer using RNA 6000 Nano assays (Agilent Technologies, Palo Alto, USA). The reverse transcription of miRNA was performed with TaqMan microRNA Reverse Transcription kit (Applied Biosystems, Foster City, CA) using stem-loop RT primers, ensuring RT efficiency and specificity [20] according to the manufacturer's instructions. The reactions were incubated for 30 min at 16°C, 30 min at 42°C, and 5 min at 85°C. The resulting cDNA was stored at −20°C until use. The reverse transcription of mRNA was performed with Transcriptor First Strand cDNA Synthesis Kit with anchored dT primers (Roche Applied Science, Indianapolis, USA) according to the manufacturer's recommendation. The reactions were incubated for 10 min at 65°C, 60 min at 50°C, and 5 min at 85°C. The resulting cDNA was stored at −20°C until use.

### 2.3. Measurement of miRNA/mRNA Expression by Quantitative RT-PCR

The gene expression for each miRNA and mRNA in BAL cells was investigated by qRT-PCR using specific primers and probes (see Table S1 and Table S2, in Supplementary Material available online at http://dx.doi.org/10.1155/2015/121378). qRT-PCR for each individual miRNA was performed in a 20 *μ*L reaction mixture that included 1.3 *μ*L of diluted RT product, 1 *μ*L of 20X TaqMan Individual microRNA assay, 10 *μ*L of 2X TaqMan Universal PCR Master Mix, No AmpErase UNG (Applied Biosystems), and 7.7 *μ*L of nuclease-free water. qRT-PCR for each individual mRNA expression was performed as described previously [21]. All reactions were performed on RotorGene3000 system (Qiagen Inc., Valencia, CA, USA); the reaction steps were as follows: 95°C for 10 min, followed by 40 cycles at 95°C for 15 s and 60°C for 1 min. The relative miRNA and mRNA expression levels were calculated by a second-derivative method (RotorGene Software 6.1.81, Corbett, Sydney, Australia); cDNA from human universal reference RNA (Stratagene, La Jolla, CA, USA) was used as a calibrator. A reference gene for miRNA analysis was endogenous control Mammalian U6 and for mRNA a reference gene* PSMB2* [21]. Changes in expression levels are presented as mean relative expression with 95% confidence interval (CI).

### 2.4. Selection of Candidate miRNAs and Identification of Binding Sites

The miRNA pathway analysis web server DIANA-mirPath v.3 (http://www.microrna.gr/miRPathv3/) [22] was used to nominate the candidate inflammation-related miRNAs based on their possible involvement in cytokine-chemokine interaction pathway. For the identification of binding sites between candidate miRNAs and mRNAs, we used the mirSystem [23] (Table S3, Supplementary Material). This web-based tool integrates the seven most often used target gene prediction algorithms: DIANA, miRanda, miRBridge, PicTar, PITA, rna22, and TargetScan. Moreover, it contains validated data of interactions between particular miRNA and its target genes from TarBase and miRecords databases.

### 2.5. Clustering Using Kohonen Self-Organizing Neural Networks

Kohonen self-organizing neural network (self-organizing maps, SOM), a clustering tool, was applied to find clusters of input data that are very close to each other [24]. The input data (the whole data set including miRNA and mRNA expression of all studied molecules) for each sarcoidosis patient were transformed to vectors, which were recorded in the neural network. Neurons in the cortex were organized in 2D; only the adjacent neurons were interconnected. If the data clustered to neurons in SOM show the internal clustering structure corresponding to the analyzed subgroups (in our case progressing and regressing disease), the data set has high potential to contain relevant biological relationships.

### 2.6. miRNA/mRNA Correlation Analysis

Correlation matrices were computed to investigate the relationships between miRNA expression and mRNA expression of* T-bet* and members of sarcoidosis-associated cytokine/chemokine-receptor network represented by Spearman correlation coefficient. The correlation matrices were graphically presented using heat maps, where a hierarchical agglomerative clustering analysis was performed to show the relationships between groups of miRNA and mRNA in the form of clusters. The colour of each cell of the heat map corresponds to the value of Spearman correlation coefficient between given miRNA and corresponding mRNA.

### 2.7. Circos Diagrams for Visualization of miRNA/mRNA Relationships

To graphically represent the relationships between miRNAs, mRNAs, and* T-bet*, a Chord diagram generated in Circos system [25] (http://mkweb.bcgsc.ca/tableviewer/) was applied for (i) sarcoidosis patients and subgroups of patients with (ii) progressing sarcoidosis and (iii) regressing sarcoidosis. In the diagram, only significant correlations are depicted (*P* < 0.05). The intensity of correlations (*w*) between individual miRNA-mRNA pairs corresponds to absolute value of Spearman correlation coefficient (*r*_*s*_), for which the mathematical transformation was performed to accentuate the differences in the intensities of correlations. For the transformation, the following formula was used:

(1) $$\begin{array}{c} w=1+{\left[\;10\times \left(\;r_{s}-t\;\right)\;\right]}^{2}, \end{array}$$

where *t* represents the threshold for the highest significant *P* value.

### 2.8. miRNA/mRNA/*T-bet* Network Analysis

For schematic representation of the relationships between miRNAs,* T-bet*, and cytokines/chemokines/receptors, we constructed weighted gene coexpression network [26]. Further, an algorithm based on analysis of the nearest neighbors between the studied molecules (represented as vertices) was applied [27]. The larger vertices (spheres) in the network have more nearest neighbors based on correlation analysis than smaller vertices. Only edges connecting the nearest neighbors (pairs with the highest correlations) were preserved. In other words, the size of the vertices (spheres) and connection among vertices show relationships between the investigated molecules in the network.

### 2.9. Statistical Analysis

Data analysis was performed using GraphPad Prism 5.01 (GraphPad Software, La Jolla, CA, USA) and SPSS 16.0 (SPSS Inc., Chicago, IL, USA). Differences in miRNA and mRNA levels between the cohorts were assessed by nonparametric Kruskal-Wallis one-way analysis-of-variance-by-ranks test; nonparametric Mann-Whitney test was utilized to determine significant differences between two groups. Spearman correlation coefficient and its corresponding *P* values were computed using R statistical software package (http://www.r-project.org/). A *P* value < 0.05 was considered significant.

## 3. Results

### 3.1. Selection of Studied miRNAs

Candidate miRNAs were selected based on their possible involvement in the regulation of inflammatory response, particularly cytokine/chemokine-receptor network. Using DIANA-mirPath v.3, we nominated 25 candidate miRNAs (*miR-let-7c, let-7d, miR-21, miR-24, miR-25*,* miR-92a*,* miR-125a*,* miR-126*,* miR-133a*,* miR-146a*,* miR-148a*,* miR-150*,* miR-155*,* miR-181a*,* miR-199a*,* miR-202*,* miR-204*,* miR-206*,* miR-212*,* miR-214*,* miR*-*222*,* miR-223*,* miR-302c*,* miR-424*, and* miR-503*) targeting cytokine/chemokine-receptor network (see Figure S1, Supplementary Material), involving many of the cytokines/chemokines and their receptors associated with sarcoidosis. A list of miRBase ID numbers and other details on studied miRNAs are stated in Table S1 (Supplementary Material); the miRNAs having (3′-UTR) “seed region” for binding to the studied mRNA are stated in Table S3 (Supplementary Material).

### 3.2. miRNA Expression Profiling

#### 3.2.1. Analysis of miRNA Expressions in Sarcoidosis Patients and Control Subjects

In order to investigate the miRNA expression pattern, the expression levels of* miR-let-7c*,* let-7d*,* miR-21*,* miR-24*,* miR-25*,* miR-92a*,* miR-125a*,* miR-126*,* miR-133a*,* miR-146a*,* miR-148a*,* miR-150*,* miR-155*,* miR-181a*,* miR-199a*,* miR-202*,* miR-204*,* miR-206*,* miR-212*,* miR-214*,* miR-222*,* miR-223*,* miR-302c*,* miR-424*, and* miR-503 *were determined in BAL cells obtained from sarcoidosis patients and control subjects.

**Figure 1:**
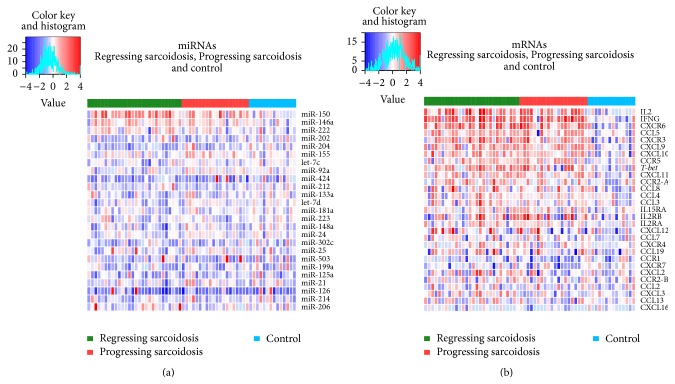
Hierarchical clustering of gene expression data. The rows represent individual (a) miRNAs and (b) mRNAs; the columns represent individual subject samples (1 column per sample) divided into three cohorts, such as sarcoidosis patients with progressing (red zone) and regressing (green zone) disease and control subjects (blue zone). The colour represents the gene expression level (blue: low expression, red: high expression); the expression levels are continuously mapped on the colour scale provided at the top of the figure. The analysis was performed using the R statistical software package (http://www.r-project.org/).

**Figure 2:**
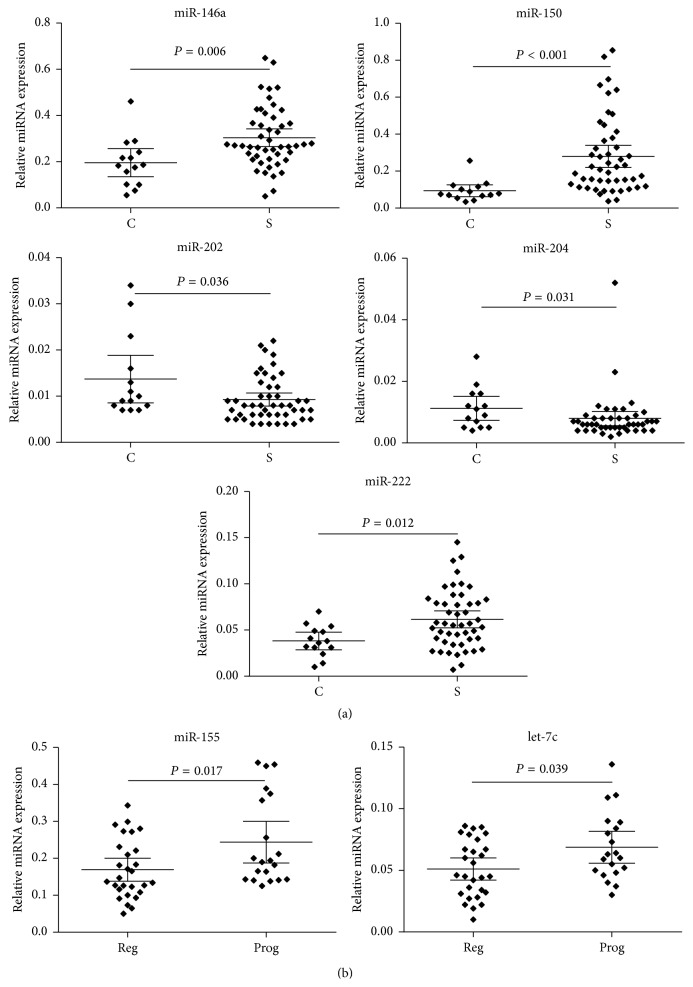
Distribution of relative miRNA expression (ratio target miRNA/reference endogenous U6 miRNA) of (a) five deregulated miRNAs between sarcoidosis patients (S) and control subjects (C) and (b) two deregulated miRNAs between patients with regressing (Reg) and progressing (Prog) sarcoidosis. Group means are indicated by horizontal bars; error bars indicate 95% CI.

When compared to control subjects, a higher number of* miR-150* (*P* < 0.001) and* miR-146a* (*P* = 0.006) (Table 2, Figure 2(a)) transcripts and lower* miR-202* (*P* = 0.036),* miR-204* (*P* = 0.031), and* miR-222* (*P* = 0.012) (Table 2, Figure 2(a)) expression were detected in sarcoidosis patients. No difference in expression levels of other studied miRNAs was observed between sarcoidosis and control subjects (Figure 1(a)).

#### 3.2.2. Analysis of miRNA Expressions in Sarcoidosis Patients with Progressing/Regressing Disease

In order to investigate the miRNA expression pattern in sarcoidosis patients subdivided according to disease outcome after 2-year follow-up, the expression levels of a set of candidate miRNAs were determined in BAL cells from progressing and regressing sarcoidosis. To exclude the notion that variations in the levels of expression of transcripts might be related to changes in cell populations, we investigated the distribution of the absolute and relative number of BAL macrophages and lymphocytes and revealed no difference between subgroups of patients with progressing and regressing sarcoidosis (*P* > 0.05).

When comparing to regressing sarcoidosis, elevated expression of* miR-155* (*P* = 0.017) and* let-7c* (*P* = 0.039) (Table 2, Figure 2(b)) was detected in progressing disease. No difference in expression levels of other studied miRNAs was observed between patients with progressing and regressing sarcoidosis (Figure 1(a)).

### 3.3. mRNA Expression Profiling

#### 3.3.1. Analysis of mRNA Expressions in Sarcoidosis Patients and Control Subjects

In order to investigate the mRNA expression pattern in sarcoidosis, the expression levels of CCL2, CCL3, CCL4, CCL5, CCL7, CCL8, CCL13, CCL19, CXCL2, CXCL3, CXCL9, CXCL10, CXCL11, CXCL12, CXCL16, CCR1, CCR2A, CCR2B, CCR5, CXCR3, CXCR4, CXCR6, CXCR7, IL2, IL2RA, IL2RB, IL15RA, IFNG, and* T-bet* were determined in BAL cells obtained from sarcoidosis patients and control subjects.

When comparing to control subjects, the expression levels of CC chemokines CCL3 (*P* = 0.043), CCL4 (*P* = 0.034), CCL5 (*P* < 0.001), and CCL8 (*P* = 0.031) and CXC chemokines CXCL9 (*P* < 0.001), CXCL10 (*P* = 0.002), and CXCL11 (*P* = 0.008) were found to be elevated in sarcoidosis patients (Table 3, Figure 1(b)). Among chemokine receptors, mRNA expressions of CCR2-var.A (*P* = 0.018), CCR5 (*P* = 0.003), CXCR3 (*P* < 0.001), and CXCR6 (*P* < 0.001) were elevated in sarcoidosis patients (Table 3, Figure 1(b)). Among cytokines/cytokine receptors, the expression levels of IL2 (*P* < 0.001), IL2RB (*P* = 0.049), IL15RA (*P* = 0.048), and IFNG (*P* < 0.001) were elevated in sarcoidosis patients (Table 3, Figure 1(b)).* T-bet* mRNA expression was also found to be elevated in sarcoidosis patients (*P* = 0.006) comparing to control subjects (Table 3, Figure 1(b)).

### 3.4. Self-Organizing Neural Networks for Sarcoidosis Patients

**Figure 3:**
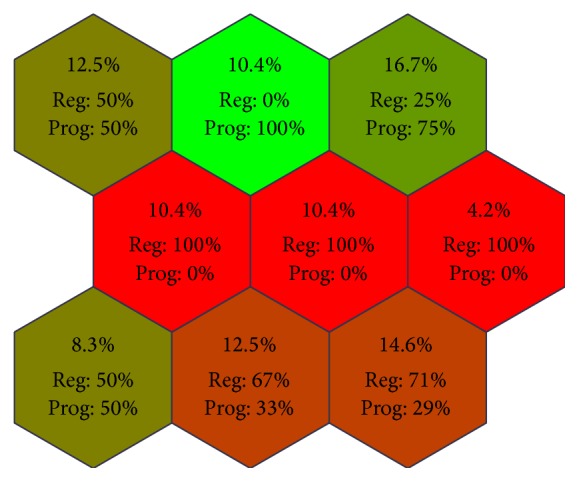
Kohonen self-organizing map (SOM) for a whole gene expression data set in BAL cells obtained from patients with sarcoidosis. Clustering shows the ratio of occurrence (given as % of patients/neuron) of two classes (progression and regression) in individual neurons (green neurons for patients with progression, red neurons for patients with regression; neurons classifying both classes are visualized as interpolation between red and green colors). The bold numbers indicate % of sarcoidosis patients clustered into particular neuron.

In order to assess the inner data structure in our data sets, we applied the Kohonen SOM. The unsupervised clustering analysis classified the sarcoidosis patients into the following clusters represented by neurons (hexagons): (1) green neurons including only patients with progression, (2) red neurons including only patients with regression; and (3) neurons including both classes visualized as interpolation between red and green colors (Figure 3).

This analysis supports the presence of inner structures in patient subgroups as assessed by disease outcome, thus supporting the fact that the subgroups differ in miRNA/mRNA profiles. Three clusters exactly matched patients with regressing sarcoidosis (25%) and one cluster patients with progressing disease (10.4%). Two clusters more or less correspond to patients with regressing sarcoidosis (27.1%), whereas one cluster more or less corresponds to patients with progressing disease (16.7%). On the other hand, two clusters of patients (20.8%) do not correspond to any class. Interestingly, the patients with regression formed more clusters than progressing patients, thus indicating that this subgroup is more heterogeneous regarding the expression profiles of studied miRNAs and mRNAs comparing to progressing disease.

### 3.5. Correlation Analysis of miRNAs,* T-bet*, and Cytokine/Chemokine-Receptor Network

In order to assess the relationships between studied miRNAs and mRNAs in BAL cells, we performed correlation analysis. To distinguish the degree of correlation between miRNA-mRNA pairs, we set up cut-off for Spearman correlation coefficient corresponding to significant *P* value (*P* < 0.05). Since no significant correlations between the studied molecules were detected in control group, the Chord diagram for this group is not presented.

**Figure 4:**
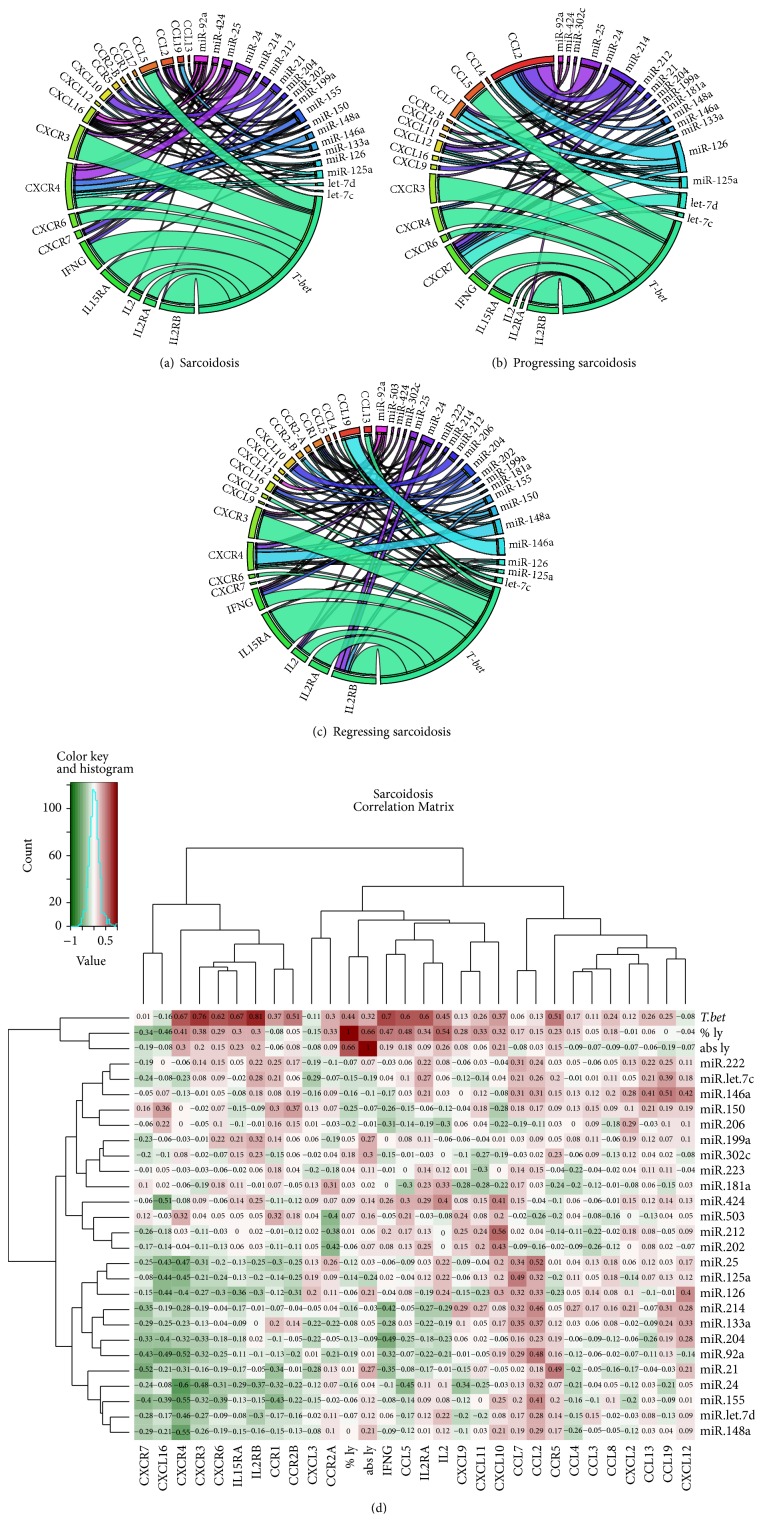
miRNA-mRNA-*T-bet* correlations in BAL cells obtained from (a) sarcoidosis patients and their subgroups with (b) progressing and (c) regressing disease are represented using Chord diagrams (circular graphs). In the Chord diagram, the intensity of the band corresponds to the significance of the correlation between particular miRNA-mRNA pair and* T-bet*-mRNA pair as assessed using Spearman's rank correlation; only significant correlations (*P* < 0.05) are visualized. (d) A hierarchical agglomerative clustering analysis presented using heat map for sarcoidosis as a whole. The colour of each cell of the heat map corresponds to value of Spearman correlation coefficient between given miRNA-mRNA pairs. % ly: % of lymphocytes in BAL fluid; abs ly: absolute number of lymphocytes/1 mL BAL fluid.

The most significant correlations in sarcoidosis as a whole were observed for the following miRNA-mRNA pairs:* miR-212*-CXCL10 (*P* < 0.001),* miR-24*-CXCR4 (*P* < 0.001),* miR-125a*-CCL7 (*P* = 0.005),* miR-146a*-CCL19 (*P* = 0.003),* miR-25*-CCL2 (*P* = 0.002),* miR-214-*CCL2 (*P* = 0.008),* miR-24*-CCL5 (*P* = 0.010),* miR-24*-CXCR3 (*P* = 0.005),* miR-21*-CXCR7 (*P* = 0.003),* miR-204*-IFNG (*P* = 0.008),* miR-148a*-CXCR4 (*P* = 0.003), and* miR-155*-CXCR4 (*P* = 0.003) (Figure 4(a)). Regarding* T-bet*, we observed correlations with CCL5 (*P* < 0.001), CXCR3 (*P* < 0.001), CXCR4 (*P* < 0.001), CXCR6 (*P* < 0.001), IL2 (*P* < 0.001), IL2RA (*P* < 0.001), IL2RB (*P* < 0.001), IL15RA (*P* < 0.001), IFNG (*P* < 0.001), CCR2B (*P* = 0.004), CXCL10 (*P* = 0.021), and CCR5 (*P* = 0.021) (Figure 4(a)).

In progressing disease, relationships for the following miRNA-mRNA pairs were observed:* miR-25-*CCL2 (*P* < 0.001),* miR-126-*CCL2 (*P* < 0.001),* miR-214-*CCL2 (*P* < 0.001),* miR-125a*-CCL7 (*P* = 0.008),* miR-126-*CCL7 (*P* = 0.009),* let-7d*-CXCR7 (*P* = 0.001), and* miR-126*-CXCR7 (*P* = 0.009) (Figure 4(b)).* T-bet* correlated with CCL5 (*P* < 0.001), CXCR3 (*P* < 0.001), CXCR4 (*P* < 0.001), IL2RB (*P* < 0.001), IL15RA (*P* < 0.001), IFNG (*P* < 0.001), CXCR6 (*P* = 0.005), CXCL10 (*P* = 0.016), CCR2B (*P* = 0.029), IL2 (*P* = 0.044), and IL2RA (*P* = 0.037) (Figure 4(b)).

In regressing sarcoidosis, we detected correlations among the following miRNA-mRNA pairs:* miR-146a*-CCL19 (*P* < 0.001),* let-7c-*CCL19 (*P* = 0.007),* miR-202*-CXCL10 (*P* = 0.010),* miR-212-*CXCL10 (*P* = 0.002),* miR-92a*-CXCL12 (*P* = 0.008),* miR-148a*-CXCR4 (*P* = 0.002),* miR-24*-IL2RB (*P* = 0.006), and* miR-25*-IL2RB (*P* = 0.006) (Figure 4(c)). Moreover,* T-bet* correlated with CXCR3 (*P* < 0.001), IL2 (*P* < 0.001), IL2RA (*P* < 0.001), IL2RB (*P* < 0.001), IL15RA (*P* < 0.001), IFNG (*P* < 0.001), CCL13 (*P* = 0.004), CXCL2 (*P* = 0.004), CXCR6 (*P* = 0.007), CXCL11 (*P* = 0.013), CCL5 (*P* = 0.018), CCR2A (*P* = 0.033), CCR2B (*P* = 0.023), and CXCR4 (*P* = 0.017) (Figure 4(c)).

In sarcoidosis patients, certain genes correlated with both* T-bet* and miRNAs simultaneously (Figure 4(d)). Correlation with both* T-bet* and miRNAs was observed for CCL5, CXCL10, CXCR3, CXCR4, CXCR6, IL2, IL15RA, and IFNG. In progressing sarcoidosis, correlation with both* T-bet* and miRNAs was observed for CXCR3 and IL2RB (Figure 4(b), Figure S2, Supplementary Material), whereas in regressing sarcoidosis* T-bet* correlated with CCL5, CCL13, CXCL11, CCR2A, CCR2B, CXCR3, CXCR4, IL2, IL2RB, IL15RA, and IFNG (Figure 4(c), Figure S2, Supplementary Material).

### 3.6. Weighted Gene Coexpression Network Analysis for miRNA-mRNA Relationships

In order to better characterize the relationships between studied miRNAs, mRNAs, and* T-bet*, we performed weighted gene coexpression network analysis.

**Figure 5:**
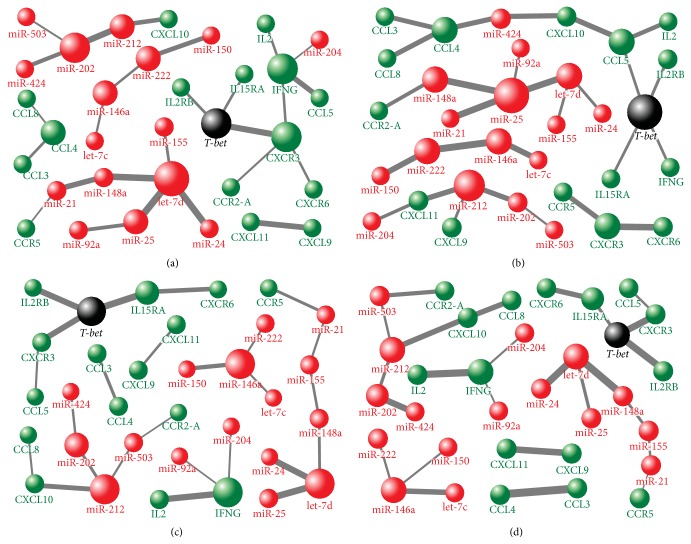
A weighted gene coexpression network analysis of miRNA-mRNA-*T-bet* relationships in BAL cells obtained from (a) sarcoidosis patients and their subgroups with (b) progressing and (c) regressing disease and from (d) control subjects. The studied molecules are represented as vertices (spheres), where larger vertices in the network have more nearest neighbors than smaller ones. Only edges connecting the nearest neighbors (pairs with the highest correlations) were preserved.

The close relationships in sarcoidosis as a whole were found between* T-bet* and IL2RB, IL15RA, and CXCR3, which was related to CXCR6 and IFNG (Figure 5(a)). IFNG related to IL2 and CCL5 and also to* miR-204*. We observed relationships also between* miR-212*-CXCL10 and* miR-21*-CCR5. Considering miRNAs, we found relationships between* let-7d, miR-155*,* miR-24*, and* miR-25* and between* miR-202*,* miR-212*,* miR-424*, and* miR-503* (Figure 5(a)).

In progressing sarcoidosis,* T-bet* related to CCL5, IL2RB, IL15RA, and IFNG (Figure 5(b)). Relationships were observed also between* miR-212* and IFNG-induced chemokines CXCL9 and CXCL11. In addition, CXCL11 related to* miR-204*. Relationships were observed also between* miR-424* and CCL4 and CXCL10, whereas* miR-148a* related to CCR2A. Among miRNAs, relationships were found between* miR-21*,* miR-25*,* miR-148a*,* miR-92a*, and* let-7d* and between* miR-146a*,* miR-222*, and* let-7c* (Figure 5(b)).

In regressing sarcoidosis, relationships were observed between* T-bet* and IL2RB, IL15RA, CXCR3, IL2, and IFNG (Figure 5(c)). We observed relationships also between* miR-212* and CXCL10 and between* miR-503* and CCR2A. Among miRNAs, relationships were detected between* let-7d, miR-148a*,* miR-24*, and* miR-25*, between* miR-146a, miR-150*,* miR-222*, and* let-7c,* and between* miR-212*,* miR-202*, and* miR-503* (Figure 5(c)).

Regarding control group, the investigated molecules were not closely related to each other (small vertices) and their relationship pattern differed from sarcoidosis-associated pattern (Figure 5(d)).

## 4. Discussion

This is the first study investigating the suggested contribution of both transcriptional and posttranscriptional control to deregulated cytokine/chemokine-receptor network in pulmonary sarcoidosis. Our correlation network analysis revealed that both inflammatory-related microRNAs and a key Th1-transcription factor* T-bet* may contribute to deregulated cytokine/chemokine-receptor network in sarcoid BAL cells, whereas contributions of transcriptional and posttranscriptional regulation differ between progressing and regressing disease as assessed by 2-year follow-up. We also for the first time investigated the gene profile of inflammation-related miRNAs in BAL cells and revealed altered miRNA pattern in BAL cells obtained from patients with sarcoidosis comparing to control subjects and also from patients with progressing versus regressing disease.

In sarcoidosis pathogenesis, the crucial role is attributed to inflammatory cytokines and chemokines, which direct circulating leukocytes to the sites of inflammation and control leukocyte activation and cytokine production, angiogenesis, and Th cell polarization [4, 28, 29]. In view of the crucial role of cytokines, chemokines, and their receptors in the pathogenesis of sarcoidosis, a fundamental question arises: how the cytokine/chemokine-receptor network is regulated. There is first evidence that cytokines and chemokines are regulated by both transcriptional and posttranscriptional mechanisms allowing for rapid and transient production in response to a variety of stimuli [30, 31]. In line with this observation, we hypothesized that the regulation of cytokine/chemokine-receptor network in BAL cells also involves coordinated participation of multiple regulatory systems, including miRNAs and a key sarcoidosis-associated Th1-transcription factor* T-bet*.

However, miRNAs pattern in BAL cells has not been investigated yet. We therefore investigated for the first time expression pattern of inflammation-related miRNAs in BAL cells and revealed downregulated* miR-202* and* miR-204* and upregulated* miR-146a*,* miR-150*, and* miR-222* expression in sarcoidosis comparing to controls. When comparing progressing to regressing sarcoidosis, a higher number of* miR-155* and* let-7c* transcripts were detected in progressing sarcoidosis. All detected miRNAs associated with sarcoidosis were already associated with regulation of inflammatory response. Inflammatory* miR-155* and* miR-146a* were found to be upregulated by NF-*κ*B [32, 33],* miR-150* involved in pulmonary inflammation [34],* miR-202* in rheumatoid arthritis [35], and* miR-204* in the production of inflammatory mediators and apoptosis [36].* MiR-222* contributed to inflammation-mediated neovessel formation [37], and* let-7c* regulated allergic airway inflammation [38]. Moreover,* miR-204* was also found to be deregulated in sarcoid lung tissues reported by Crouser et al. [9]. Interestingly, when comparing the miRNA profile in sarcoid BAL cells obtained in this study with profile reported in sarcoid lung tissues [9], we obtained different pattern of top-deregulated miRNAs (*miR-92a, miR-125a, miR-126, miR-181a, miR-206*, and* miR-302*) found in sarcoid lung tissues, thus supporting the fact that miRNA expression signatures vary depending on the tissues and cell types examined.

However, it is still unclear how miRNAs regulate cytokines, because 3′-untranslated region (3′-UTR) of most cytokines lacks critical “seed region” for binding to mRNA [39]. Current studies showed that cytokine and other immune genes may be regulated by other elements (e.g., ARE machinery components) [40] or through highly biologically functional “nonseed” miRNA target sites, containing single mismatches, GU wobbles, insertions, or deletions in the seed-match regions [41–43]. These “nonseed” miRNA target sites represent up to 50% of all miRNA-mRNA interactions and remain uncovered, despite their functional significance, using current target prediction algorithms [39]. Importantly, miRNA regulation may not be directly at the cytokine/chemokine or receptor level but rather at upstream or downstream steps in the pathway. These facts may explain our observation of correlation between numerous miRNAs and cytokine expression (e.g., CXCR4 and* miR-24*), where no binding site was discovered by prediction algorithms as well as numerous positive correlations between miRNAs and cytokine and chemokine expression (e.g., CCL2 and* miR-214*) as reported also in other studies [44, 45]. Moreover, the unique role of miRNAs in inflammation is that the miRNAs modulate the expression of target genes to an optimum level rather than participating in on/off decisions [40].

Besides posttranscriptional regulation by miRNAs, also transcriptional control by transcription factors may contribute to deregulated cytokine/chemokine-receptor network in pulmonary sarcoidosis. We recently reported crucial role for a Th1-transcription factor* T-bet*, which has emerged as key regulator of IFNG and chemokine receptor CXCR3 and other immune genes in sarcoid inflammation [12]. In the present study, we searched for evidence of possible participation of transcriptional and posttranscriptional regulation by miRNAs and* T-bet* in controlling the inflammation in sarcoid BAL cells. Our correlation analysis revealed relationship of* T-bet* and sarcoid inflammation, namely, its association with key sarcoidosis-associated cytokine IFNG, and receptors IL2RB, IL15RA, CXCR3, and CXCR6, regardless of the disease outcome.

Importantly, the relationships between* T-bet* and particular miRNAs regarding the chemokine network changed between subgroups of patients, who differed in disease outcome after 2-year follow-up. In progressing sarcoidosis,* T-bet* correlated with CCL5, CXCR4, and CXCR7. We also observed positive correlations between numerous miRNAs and expression of CCL2, CCL7, and CXCL12 and negative correlation with CCL5, CXCR3, CXCR4, and CXCR7 expression in progressing disease. In regressing disease,* T-bet* showed less pronounced correlation with IL2 and receptors CXCR4, CXCR7, and IL2RA than in progression. Also miRNA pattern changed; in regression, the correlation analysis revealed positive correlation between* miR-146a* and CCL19 as well as* miR-148a* and CXCR4. Our analysis therefore nominated CCL2 and CCL5, both chemoattractants of mononuclear and mast cells [46], as well as CCL19, a chemokine implicated in T-lymphocyte recruitment [47], as key candidate chemokines associated with prognosis of pulmonary sarcoidosis. Similarly, CXCR4 and CXCR7 and their ligand, CXCL12, playing a role in regulation of leukocyte mobilization and trafficking [48, 49], deserve further investigation.

To better characterize the network of inflammation-related miRNAs,* T-bet*, and cytokine/chemokine-receptor network in sarcoid BAL cells, we performed weighted correlation network analysis. This analysis further supported the crucial relationships between* T-bet*, CCL5, IFNG, IL2RB, and IL15RA, together with* let-7d* and* miR-202* in progressing sarcoidosis. In regressing disease,* T-bet* was more closely related to CXCR3 and less to IFNG and CCL5 than in progressing disease. Regarding miRNAs in regressing disease,* miR-212*,* miR-146a*, and* let-7d* were highlighted. Given the complexity of miRNA-mRNA-transcription factor network, the understanding of the contribution of network members and regulatory mechanisms is intricate and requires further studies which may reveal the functional outcome of the interactions between the associated molecules.

Our study has several limitations. First, this exploratory study on miRNA and mRNA expression pattern and their relationships was performed in unseparated BAL cells. To obtain complete picture of the regulatory processes ongoing in sarcoid inflammation, this study should continue by analysing the miRNA/mRNA expressions in distinct cell subpopulations. We are also aware that our set of inflammation-related miRNAs and chemokine/receptor genes, selected based on their involvement in the pathogenesis of sarcoidosis, does not cover the whole potential network of molecules/pathways contributing to this disease. Further, we did not perform the functional studies needed to confirm the biological relevance of the obtained relationships between* T-bet*, miRNAs, and chemokine network. However, we believe that our study may contribute to nomination of interesting relationships for future studies of regulatory mechanisms involved in the pathogenesis of pulmonary sarcoidosis and its phenotypes.

## 5. Conclusions

Our correlation network analysis implies both microRNAs and Th1-transcription factor* T-bet* in the regulation of cytokine/chemokine-receptor network in BAL cells in sarcoidosis. Future functional studies are however needed to confirm the biological relevance of the obtained relationships. The correlation analysis showed more pronounced regulatory capability of* T-bet* to sarcoidosis-associated chemokine receptors and cytokines than miRNAs, which function rather as “fine-tuners” of cytokine/chemokine gene expression. Moreover, we reported altered miRNA pattern in BAL cells obtained from sarcoidosis patients as well as from subgroups of patients with progressing and regressing sarcoidosis as assessed by 2-year follow-up. The knowledge and understanding of the regulatory mechanisms underlying the abnormal inflammatory response in sarcoid lungs could shed light on the cause and progression of sarcoidosis and many inflammatory and autoimmune diseases.

## Supplementary Material

Correlation network analysis reveals relationships between microRNAs, transcription factor *T-bet* and deregulated cytokine/chemokine-receptor network in pulmonary sarcoidosis

## Figures and Tables

**Table 1:** Clinical and laboratory data of enrolled patients with pulmonary sarcoidosis.

Characteristics	Sarcoidosis	Regression	Progression	Controls
	(*N* = 48)	(*N* = 28)	(*N* = 20)	(*N* = 14)
Age, yrs	47.9 (29–72)	45.8 (29–70)	50.9 (32–72)	39.6 (19–63)
Sex (male/female)	23/25	12/16	11/9	9/5
Smoking (y/n/ex)	0/37/11	0/20/8	0/17/3	0/9/5
Pulmonary/pulmonary plus extrapulmonary involvement	35/13	18/10	17/3	—
CXR stages (I/II)	15/33	10/18	5/15	—
Löfgren's syndrome (y/n)	7/41	4/24	3/17	—
BALF differential count^†^				
% macrophages	75.6 ± 5.8 (40.0–94.0)	74.3 ± 6.6 (40.0–94.0)	77.5 ± 4.5 (60.5–87.4)	92.2 ± 13.5 (83.8–97.4)
% lymphocytes	21.6 ± 5.5 (4.6–49.0)	22.8 ± 6.2 (6.0–49.0)	19.9 ± 4.6 (4.6–36.0)	6.2 ± 2.3 (1.6–11.0)
% neutrophils	2.1 ± 1.6 (0.0–18.0)	2.3 ± 1.8 (0.0–18.0)	1.8 ± 1.2 (0.0–10.0)	1.5 ± 0.8 (0.3–6.0)
% eosinophils	1.3 ± 1.9 (0.0–17.3)	1.3 ± 1.6 (0.0–17.0)	1.4 ± 1.9 (0.0–17.3)	0.3 ± 0.1 (0.3–0.6)
% CD3+^#^	83.6 ± 6.4 (48.0–98.0)	83.8 ± 7.1 (48.0–96.0)	83.3 ± 5.7 (54.0–98.0)	74.1 ± 7.2 (40.0–92.0)
% CD4+^#^	64.9 ± 8.8 (23.0–92.0)	66.9 ± 8.9 (27.0–92.0)	62.0 ± 8.9 (23.0–86.0)	45.7 ± 7.3 (22.0–67.0)
% CD8+^#^	16.8 ± 5.7 (2.0–50.0)	14.4 ± 4.9 (2.0–42.0)	20.3 ± 6.4 (4.0–50.0)	27.4 ± 5.5 (14.0–51.0)
% CD19+^#^	1.03 ± 1.2 (0.0–14.0)	0.9 ± 1.3 (0.0–14.0)	1.2 ± 1.1 (0.0–8.0)	1.2 ± 0.6 (0.0–3.0)
BALF CD4+/CD8+ ratio	6.8 ± 3.6 (0.8–46.0)	7.9 ± 4.2 (1.1–46.0)	5.3 ± 2.6 (0.8–21.3)	2.0 ± 0.5 (0.5–3.7)

BALF: bronchoalveolar lavage fluid; *N*: number of patients; n: no; y: yes; ex: ex-smoker; CXR: chest X-ray; —: not relevant.

Data are presented as mean ± SD (minimum and maximum in parentheses).

^†^Data were not available for four patients.

^#^% of CD3, CD4, CD8, and CD19 refers to total lymphocyte counts.

**Table 2:** Comparison of miRNA expression profiles between sarcoidosis (S) patients, subdivided according to the outcome after 2-year follow-up (Reg: regression; Prog: progression), and control subjects (C). All the data are presented as Mean Expression Level (95% confidence interval). Results are expressed relative to levels of endogenous U6 miRNA. *N*: number of patients. Bold font indicates significant *P* value.

	S	Reg S	Prog S	C	*P*	*P*	*P*
	(*N* = 48)	(*N* = 28)	(*N* = 20)	(*N* = 14)	(Kruskal-Wallis)	S versus C	Reg versus Prog
miR-let-7c-5p	0.058 (0.051–0.066)	0.051 (0.042–0.060)	0.069 (0.056–0.082)	0.069 (0.052–0.087)	0.127	0.239	**0.039**

miR-let-7d-5p	0.136 (0.118–0.154)	0.124 (0.100–0.147)	0.153 (0.126–0.181)	0.155 (0.120–0.190)	0.250	0.256	0.103

miR-21-5p	2.022 (1.618–2.427)	1.676 (1.300–2.051)	2.508 (1.693–3.323)	2.023 (1.253–2.794)	0.585	0.833	0.161

miR-24-3p	0.522 (0.462–0.582)	0.488 (0.409–0.567)	0.570 (0.473–0.667)	0.571 (0.432–0.711)	0.432	0.405	0.174

miR-25-3p	0.110 (0.071–0.150)	0.117 (0.050–0.184)	0.101 (0.077–0.124)	0.089 (0.042–0.137)	0.485	0.434	0.191

miR-92a-3p	0.364 (0.312–0.416)	0.319 (0.255–0.384)	0.421 (0.336–0.506)	0.362 (0.272–0.452)	0.277	0.814	0.061

miR-125a-5p	0.044 (0.034–0.054)	0.046 (0.029–0.062)	0.042 (0.031–0.052)	0.058 (0.020–0.096)	0.982	0.814	0.770

miR-126-3p	0.054 (0.005–0.102)	0.077 (0.007–0.160)	0.021 (0.012–0.030)	0.057 (0.004–0.118)	0.992	0.860	0.810

miR-133a-3p	0.012 (0.010–0.015)	0.012 (0.008–0.015)	0.013 (0.009–0.017)	0.014 (0.010–0.018)	0.589	0.248	0.557

miR-146a-5p	0.304 (0.266–0.342)	0.294 (0.235–0.352)	0.319 (0.271–0.366)	0.196 (0.135–0.257)	**0.020**	**0.006**	0.331

miR-148a-3p	0.061 (0.050–0.072)	0.056 (0.042–0.069)	0.068 (0.049–0.087)	0.074 (0.050–0.097)	0.507	0.342	0.268

miR-150-5p	0.280 (0.219–0.340)	0.277 (0.199–0.355)	0.283 (0.179–0.386)	0.093 (0.061–0.125)	**<0.001**	**<0.001**	0.983

miR-155-5p	0.200 (0.170–0.231)	0.169 (0.138–0.200)	0.244 (0.187–0.300)	0.168 (0.126–0.210)	0.111	0.550	**0.017**

miR-181a-5p	0.106 (0.091–0.121)	0.102 (0.084–0.120)	0.112 (0.085–0.139)	0.122 (0.091–0.153)	0.650	0.263	0.630

miR-199a-5p	0.012 (0.010–0.014)	0.011 (0.008–0.014)	0.013 (0.009–0.016)	0.016 (0.007–0.024)	0.919	0.748	0.532

miR-202-3p	0.009 (0.008–0.011)	0.010 (0.007–0.013)	0.013 (0.006–0.020)	0.014 (0.009–0.019)	0.173	**0.036**	0.564

miR-204-5p	0.008 (0.006–0.010)	0.006 (0.005–0.007)	0.010 (0.005–0.015)	0.011 (0.007–0.015)	**0.030**	**0.031**	0.061

miR-206	0.026 (0.002–0.064)	0.039 (0.003–0.101)	0.010 (0.001–0.021)	0.005 (0.003–0.007)	0.999	0.928	0.963

miR-212-3p	0.010 (0.008–0.013)	0.010 (0.008–0.012)	0.011 (0.006–0.017)	0.012 (0.009–0.016)	0.490	0.158	0.705

miR-214-3p	0.009 (0.006–0.011)	0.008 (0.006–0.010)	0.010 (0.005–0.014)	0.010 (0.005–0.014)	0.968	0.866	0.621

miR-222-3p	0.038 (0.029–0.048)	0.059 (0.047–0.072)	0.065 (0.050–0.080)	0.062 (0.052–0.071)	0.059	**0.012**	0.668

miR-223-3p	0.808 (0.679–0.936)	0.815 (0.615–1.014)	0.798 (0.644–0.951)	0.901 (0.695–1.106)	0.692	0.270	0.826

miR-302c-3p	0.061 (0.048–0.074)	0.060 (0.041–0.079)	0.065 (0.046–0.083)	0.089 (0.034–0.143)	0.820	0.427	0.631

miR-424-5p	0.103 (0.002–0.289)	0.013 (0.002–0.025)	0.009 (0.006–0.012)	0.018 (0.005–0.031)	0.426	0.143	0.905

miR-503-5p	0.038 (0.030–0.046)	0.041 (0.030–0.052)	0.035 (0.023–0.046)	0.058 (0.022–0.094)	0.480	0.643	0.112

**Table 3:** Comparison of mRNA expression profiles of studied cytokines, chemokines, chemokine receptors, and *T-bet* between sarcoidosis (S) patients, subdivided according to the outcome after 2-year follow-up (Reg: regression; Prog: progression), and control subjects (C). All the data are presented as Mean Expression Level (95% confidence interval). Results are expressed relative to levels of a housekeeping gene PSMB2. *N*: number of patients. Bold font indicates significant *P* value.

	S	Reg S	Prog S	C	*P*	*P*	*P*
	(*N* = 48)	(*N* = 28)	(*N* = 20)	(*N* = 14)	(Kruskal-Wallis)	C versus S	Reg versus Prog
CCL2	0.058 (0.051–0.066)	0.051 (0.042–0.060)	0.069 (0.056–0.082)	0.069 (0.052–0.087)	0.950	0.602	0.875

CCL3	0.136 (0.118–0.154)	0.124 (0.100–0.147)	0.153 (0.126–0.181)	0.155 (0.120–0.190)	0.110	**0.043**	0.237

CCL4	2.022 (1.618–2.427)	1.676 (1.300–2.051)	2.508 (1.693–3.323)	2.023 (1.253–2.794)	0.157	**0.034**	0.746

CCL5	0.522 (0.462–0.582)	0.488 (0.409–0.567)	0.570 (0.473–0.667)	0.571 (0.432–0.711)	**0.002**	**<0.001**	0.834

CCL7	0.110 (0.071–0.150)	0.117 (0.050–0.184)	0.101 (0.077–0.124)	0.089 (0.042–0.137)	0.500	0.152	1.000

CCL8	0.364 (0.312–0.416)	0.319 (0.255–0.384)	0.421 (0.336–0.506)	0.362 (0.272–0.452)	0.147	**0.031**	1.000

CCL13	0.044 (0.034–0.054)	0.046 (0.029–0.062)	0.042 (0.031–0.052)	0.058 (0.020–0.096)	0.861	0.939	0.420

CCL19	0.054 (0.005–0.102)	0.077 (0.007–0.160)	0.021 (0.012–0.030)	0.057 (0.004–0.118)	0.477	0.216	0.403

CXCL2	0.012 (0.010–0.015)	0.012 (0.008–0.015)	0.013 (0.009–0.017)	0.014 (0.010–0.018)	0.429	0.414	0.161

CXCL3	0.304 (0.266–0.342)	0.294 (0.235–0.352)	0.319 (0.271–0.366)	0.196 (0.135–0.257)	0.069	0.608	**0.011**

CXCL9	0.061 (0.050–0.072)	0.056 (0.042–0.069)	0.068 (0.049–0.087)	0.074 (0.050–0.097)	**0.005**	**<0.001**	0.670

CXCL10	0.280 (0.219–0.340)	0.277 (0.199–0.355)	0.283 (0.179–0.386)	0.093 (0.061–0.125)	**0.009**	**0.002**	0.925

CXCL11	0.200 (0.170–0.231)	0.169 (0.138–0.200)	0.244 (0.187–0.300)	0.168 (0.126–0.210)	**0.045**	**0.008**	0.754

CXCL12	0.106 (0.091–0.121)	0.102 (0.084–0.120)	0.112 (0.085–0.139)	0.122 (0.091–0.153)	0.180	0.101	0.204

CXCL16	0.012 (0.010–0.014)	0.011 (0.008–0.014)	0.013 (0.009–0.016)	0.016 (0.007–0.024)	0.241	0.982	0.612

CCR1	0.009 (0.008–0.011)	0.010 (0.007–0.013)	0.013 (0.006–0.020)	0.014 (0.009–0.019)	0.289	0.277	0.112

CCR2-var.A	0.008 (0.006–0.010)	0.006 (0.005–0.007)	0.010 (0.005–0.015)	0.011 (0.007–0.015)	0.051	**0.018**	0.241

CCR2-var.B	0.026 (0.002–0.064)	0.039 (0.003–0.101)	0.010 (0.001–0.021)	0.005 (0.003–0.007)	0.779	0.444	0.587

CCR5	0.010 (0.008–0.013)	0.010 (0.008–0.012)	0.011 (0.006–0.017)	0.012 (0.009–0.016)	**0.016**	**0.003**	0.427

CXCR3	0.009 (0.006–0.011)	0.008 (0.006–0.010)	0.010 (0.005–0.014)	0.010 (0.005–0.014)	**0.002**	**<0.001**	0.691

CXCR4	0.038 (0.029–0.048)	0.059 (0.047–0.072)	0.065 (0.050–0.080)	0.062 (0.052–0.071)	0.295	0.152	0.271

CXCR6	0.808 (0.679–0.936)	0.815 (0.615–1.014)	0.798 (0.644–0.951)	0.901 (0.695–1.106)	**<0.001**	**<0.001**	0.616

CXCR7	0.061 (0.048–0.074)	0.060 (0.041–0.079)	0.065 (0.046–0.083)	0.089 (0.034–0.143)	0.667	0.392	0.417

IL2	0.103 (0.002–0.289)	0.013 (0.002–0.025)	0.009 (0.006–0.012)	0.018 (0.005–0.031)	**<0.001**	**<0.001**	0.730

IL2RA	0.038 (0.030–0.046)	0.041 (0.030–0.052)	0.035 (0.023–0.046)	0.058 (0.022–0.094)	0.210	0.051	0.730

IL2RB	0.038 (0.029–0.048)	0.059 (0.047–0.072)	0.065 (0.050–0.080)	0.062 (0.052–0.071)	0.131	**0.049**	0.272

IL15RA	0.808 (0.679–0.936)	0.815 (0.615–1.014)	0.798 (0.644–0.951)	0.901 (0.695–1.106)	0.182	**0.048**	0.530

IFNG	0.061 (0.048–0.074)	0.060 (0.041–0.079)	0.065 (0.046–0.083)	0.089 (0.034–0.143)	**<0.001**	**<0.001**	0.565

*T-bet*	0.103 (0.002–0.289)	0.013 (0.002–0.025)	0.009 (0.006–0.012)	0.018 (0.005–0.031)	**0.031**	**0.006**	0.573

## Abbreviations of Investigated Molecules

CCL2/MCP-1: Chemokine (C-C motif) ligand 2/monocyte chemoattractant protein-1
CCL3/MIP-1A: Chemokine (C-C motif) ligand 3/macrophage inflammatory protein-1 alpha
CCL4/MIP-1B: Chemokine (C-C motif) ligand 4/macrophage inflammatory protein-1 beta
CCL5/RANTES: Chemokine (C-C motif) ligand 5/regulated upon activation, normally T-expressed, and presumably secreted
CCL7/MCP-3: Chemokine (C-C motif) ligand 7/monocyte chemoattractant protein-3
CCL8/MCP-2: Chemokine (C-C motif) ligand 8/monocyte chemoattractant protein-2
CCL13/MCP-4: Chemokine (C-C motif) ligand 13/monocyte chemoattractant protein-4
CCL19/MIP-3B: Chemokine (C-C motif) ligand 19/macrophage inflammatory protein-3 beta
CXCL2/MIP-2A: Chemokine (C-X-C motif) ligand 2/macrophage inflammatory protein-2 alpha
CXCL3/MIP-2B: Chemokine (C-X-C motif) ligand 3/macrophage inflammatory protein-2 beta
CXCL9/MIG: Chemokine (C-X-C motif) ligand 9/monokine induced by gamma interferon
CXCL10/IP-10: Chemokine (C-X-C motif) ligand 10/interferon gamma-induced protein-10
CXCL11/I-TAC: Chemokine (C-X-C motif) ligand 11/interferon-inducible T-cell alpha chemoattractant
CXCL12/SDF-1: Chemokine (C-X-C motif) ligand 12/stromal cell-derived factor-1
CXCL16/SR-PSOX: Chemokine (C-X-C motif) ligand 16/scavenger receptor for phosphatidylserine and oxidized low density lipoprotein
CCR1: Chemokine (C-C motif) receptor 1
CCR2A: Chemokine (C-C motif) receptor 2, transcript variant A
CCR2B: Chemokine (C-C motif) receptor 2, transcript variant B
CCR5: Chemokine (C-C motif) receptor 5
CXCR3: Chemokine (C-X-C motif) receptor 3
CXCR4: Chemokine (C-X-C motif) receptor 4
CXCR6: Chemokine (C-X-C motif) receptor 6
CXCR7: Chemokine (C-X-C motif) receptor 7
IL2: Interleukin 2
IL2RA: Interleukin 2 receptor, alpha
IL2RB: Interleukin 2 receptor, beta
IL15RA: Interleukin 15 receptor, alpha
IFNG: Interferon gamma
PSMB2: Proteasome (prosome, macropain) subunit, beta type, 2
*T-bet*: T-box 21, T-cell-specific T-box transcription factor.
