# Supplementary material for: Correlation Network Analysis Reveals Relationships between MicroRNAs, Transcription Factor T-bet, and Deregulated Cytokine/Chemokine-Receptor Network in Pulmonary Sarcoidosis
Source: Mediators Inflamm. 2015 Nov 30;2015:121378. doi: 10.1155/2015/121378 (PMC4677216; doi:10.1155/2015/121378)

***On-line supplement***

**Correlation network analysis reveals relationships between microRNAs, transcription factor *T-bet* and deregulated cytokine/chemokine-receptor network in pulmonary sarcoidosis**

Dyskova Tereza^*^, Fillerova Regina^*^, Novosad Tomas, Kudelka Milos, Zurkova Monika, Gajdos Petr, Kolek Vitezslav, Kriegova Eva

**Table S1**. A list of candidate miRNAs, their sequences, miRBase Accession numbers and miRNA Assay IDs. See the following website for individual miRNA Assay details: <http://www6.appliedbiosystems.com/cms/groups/cms_052133.xls>.

| **ID** | **sequence** | **miRBase Accession Number** | **MiRNA Assay ID** |
| --- | --- | --- | --- |
| hsa-let-7c-5p | UGAGGUAGUAGGUUGUAUGGUU | MIMAT0000064 | 000379 |
| hsa-let-7d-5p | AGAGGUAGUAGGUUGCAUAGUU | MIMAT0000065 | 002283 |
| hsa-miR-21-5p | UAGCUUAUCAGACUGAUGUUGA | MIMAT0000076 | 000397 |
| hsa-miR-24-3p | UGGCUCAGUUCAGCAGGAACAG | MIMAT0000080 | 000402 |
| hsa-miR-25-3p | CAUUGCACUUGUCUCGGUCUGA | MIMAT0000081 | 000403 |
| hsa-miR-92a-3p | UAUUGCACUUGUCCCGGCCUG | MIMAT0000092 | 000431 |
| hsa-miR-125a-5p | UCCCUGAGACCCUUUAACCUGUG | MIMAT0000443 | 002198 |
| hsa-miR-126-3p | UCGUACCGUGAGUAAUAAUGC | MIMAT0000445 | 002228 |
| hsa-miR-133a-3p | UUUGGUCCCCUUCAACCAGCUG | MIMAT0000427 | 002246 |
| hsa-miR-146a-5p | UGAGAACUGAAUUCCAUGGGUU | MIMAT0000449 | 000468 |
| hsa-miR-148a-3p | UCAGUGCACUACAGAACUUUGU | MIMAT0000243 | 000470 |
| hsa-miR-150-5p | UCUCCCAACCCUUGUACCAGUG | MIMAT0000451 | 000473 |
| hsa-miR-155-5p | UUAAUGCUAAUCGUGAUAGGGG | MIMAT0000646 | 002623 |
| hsa-miR-181a-5p | AACAUUCAACGCUGUCGGUGAGU | MIMAT0000256 | 000480 |
| hsa-miR-199a-5p | CCCAGUGUUCAGACUACCUGUUC | MIMAT0000231 | 000498 |
| hsa-miR-202-3p | AGAGGUAUAGGGCAUGGGAA | MIMAT0002811 | 002363 |
| hsa-miR-204-5p | UUCCCUUUGUCAUCCUAUGCCU | MIMAT0000265 | 000508 |
| hsa-miR-206 | UGGAAUGUAAGGAAGUGUGUGG | MIMAT0000462 | 000510 |
| hsa-miR-212-3p | UAACAGUCUCCAGUCACGGCC | MIMAT0000269 | 000515 |
| hsa-miR-214-3p | ACAGCAGGCACAGACAGGCAG | MIMAT0000271 | 002306 |
| hsa-miR-222-3p | AGCUACAUCUGGCUACUGGGU | MIMAT0000279 | 002276 |
| hsa-miR-223-3p | UGUCAGUUUGUCAAAUACCCCA | MIMAT0000280 | 002295 |
| hsa-miR-302c-3p | UAAGUGCUUCCAUGUUUCAGUGG | MIMAT0000717 | 000533 |
| hsa-miR-424-5p | CAGCAGCAAUUCAUGUUUUGAA | MIMAT0001341 | 000604 |
| hsa-miR-503-5p | UAGCAGCGGGAACAGUUCUGCAG | MIMAT0002874 | 001048 |

**Table S2.** Description of investigated genes and used primers; LNA probes and amplicon sizes in reverse transcriptase-polymerase chain reaction.

| Gene abbreviation | Gene name (synonyms) | GenBank* Accession number | Sense, antisense primers | LNA probe^¥^ |
| --- | --- | --- | --- | --- |
| CCL2 | chemokine (C-C motif) ligand 2/MCP-1 | [NM_002982.](https://qpcr1.probefinder.com/showsequence.jsp;jsessionid=A2F3B7C5545C6D8577FDC8DEBA2C7D55?BiologyID=A2F3B7C5545C6D8577FDC8DEBA2C7D55141611)3 | 5´-agtctctgccgcccttct-3´  5´-gtgactggggcattgattg-3´ | #40 |
| CCL3 | chemokine (C-C motif) ligand 3/MIP-1A | [NM_002983.2](https://qpcr.probefinder.com/showsequence.jsp;jsessionid=0DA0BE9FECA4BDDDFA57D1B182637E50.worker2?seqNo=889838743) | 5´-cagaatcatgcaggtctccac-3´  5´-gcgtgtcagcagcaagtg-3´ | #56 |
| CCL4 | chemokine (C-C motif) ligand 4/MIP-1B | [NM_002984.2](https://qpcr.probefinder.com/showsequence.jsp;jsessionid=B049628F43C2CD28540E5844C011ABAF.worker5?seqNo=1583422786) | 5´-ctctccagcgctctcagc-3´  5´-accacaaagttgcgaggaag-3´ | #40 |
| CCL5 | chemokine (C-C motif) ligand 5/RANTES | [NM_002985.](https://qpcr1.probefinder.com/showsequence.jsp;jsessionid=323E5EE0893606ED32B7DC66E2B2971B?BiologyID=323E5EE0893606ED32B7DC66E2B2971B134141)2 | 5´-cctcattgctactgccctct-3´  5´-ggtgtggtgtccgaggaata-3´ | #16 |
| CCL7 | chemokine (C-C motif) ligand 7/MCP-3 | [NM_006273.2](https://qpcr.probefinder.com/showsequence.jsp;jsessionid=B049628F43C2CD28540E5844C011ABAF.worker5?seqNo=62914585) | 5´-gaaagcctctgcagcacttc-3´  5´-aatctgtagcagcaggtagttgaa-3´ | #84 |
| CCL8 | chemokine (C-C motif) ligand 8/MCP-2 | [NM_005623.2](https://qpcr.probefinder.com/showsequence.jsp;jsessionid=B049628F43C2CD28540E5844C011ABAF.worker5?seqNo=1775141609) | 5´-ccctcagggacttgctcag-3´  5´-tctccagcctctggatagga-3´ | #62 |
| CCL13 | chemokine (C-C motif) ligand 13/MCP-4 | [NM_005408.2](https://qpcr.probefinder.com/showsequence.jsp;jsessionid=B049628F43C2CD28540E5844C011ABAF.worker5?seqNo=1568405816) | 5´-accttcaacatgaaagtctctgc-3´  5´-ggacgttgagtgcatctgg-3´ | #40 |
| CCL19 | chemokine (C-C motif) ligand 19/MIP-3B | [NM_006274.2](https://qpcr.probefinder.com/showsequence.jsp;jsessionid=B049628F43C2CD28540E5844C011ABAF.worker5?seqNo=2007569785) | 5´-gcctctgttcaccctccat-3´  5´-agcatcattggtgccactc-3´ | #60 |
| CXCL2 | chemokine (C-X-C motif) ligand 2/MIP-2A | [NM_002089.3](https://qpcr.probefinder.com/showsequence.jsp;jsessionid=0DA0BE9FECA4BDDDFA57D1B182637E50.worker2?seqNo=1025673318) | 5´-cccatggttaagaaaatcatcg-3´  5´-cttcaggaacagccaccaat-3´ | #69 |
| CXCL3 | chemokine (C-X-C motif) ligand 3/MIP-2B | [NM_002090.2](https://qpcr.probefinder.com/showsequence.jsp;jsessionid=B049628F43C2CD28540E5844C011ABAF.worker5?seqNo=708277785) | 5´-aaatcatcgaaaagatactgaacaag-3´  5´-ggtaagggcagggaccac-3´ | #04 |
| CXCL9 | chemokine (C-X-C motif) ligand 9/MIG | [NM_002416.](https://qpcr1.probefinder.com/showsequence.jsp;jsessionid=D0D4116CCCEE82B05F0F93EBB2B90F23?BiologyID=D0D4116CCCEE82B05F0F93EBB2B90F23141141)1 | 5´-ccttaaacaatttgccccaag-3´  5´-ttgaactccattcttcagtgtagc-3´ | #04 |
| CXCL10 | chemokine (C-X-C motif) ligand 10/IP-10 | [NM_001565.](https://qpcr1.probefinder.com/showsequence.jsp;jsessionid=D0D4116CCCEE82B05F0F93EBB2B90F23?BiologyID=D0D4116CCCEE82B05F0F93EBB2B90F23141141)1 | 5´-gaaagcagttagcaaggaaatgt-3´  5´-gacatatactccatgtagggaagtga-3´ | #34 |
| CXCL11 | chemokine (C-X-C motif) ligand 11/I-TAC | [NM_005409.](https://qpcr1.probefinder.com/showsequence.jsp;jsessionid=D0D4116CCCEE82B05F0F93EBB2B90F23?BiologyID=D0D4116CCCEE82B05F0F93EBB2B90F23141141)3 | 5´-ttggctgtgatattgtgtgct-3´  5´-atgcaaagacagcgtcctct-3´ | #60 |
| CXCL12 | chemokine (C-X-C motif) ligand 12/SDF-1 | [NM_199168.3](https://qpcr.probefinder.com/showsequence.jsp;jsessionid=B049628F43C2CD28540E5844C011ABAF.worker5?seqNo=151919708) | 5´-gctggtcctcgtgctgac-3´  5´-gcatgggcatctgtagctc-3´ | **#13** |
| CXCL16 | chemokine (C-X-C motif) ligand 16/SR-PSOX | [NM_022059.2](https://qpcr.probefinder.com/showsequence.jsp;jsessionid=B049628F43C2CD28540E5844C011ABAF.worker5?seqNo=1554589059) | 5´-tgagagcttaccatcggtgtc-3´  5´-agcatgtccacattctttgaga-3´ | **#17** |
| CCR1 | chemokine (C-C motif) receptor 2 | [NM_001295.2](http://www.ncbi.nlm.nih.gov/entrez/viewer.fcgi?val=NM_001295.2) | Hs00174298_m1** |  |
| CCR2-var.A | chemokine (C-C motif) receptor 2, transcript variant A | NM_000647.4 | 5´-cccatcatctatgccttcgt**-3**  **5´-**ggcaatcctacagccaagag**-3´** | **#07** |
| CCR2-var.B | chemokine (C-C motif) receptor 2, transcript variant B | NM_000648.2 | **5´-**tgagacaagccacaagctga**-3´**  **5´-**ttctgataaaccgagaacgagat**-3´** | **#56** |
| CCR5 | chemokine (C-C motif) receptor 5 | [NM_001100168.1](http://www.ncbi.nlm.nih.gov/entrez/viewer.fcgi?val=NM_001100168.1)  [NM_000579.3](http://www.ncbi.nlm.nih.gov/entrez/viewer.fcgi?val=NM_000579.3) | Hs00152917_m1** |  |
| CXCR3 | chemokine (C-X-C motif) receptor 3 | [NM_001504.1](http://qpcr.probefinder.com/MainServlet.do) | Hs00171041_m1** |  |
| CXCR4 | chemokine (C-X-C motif) receptor 4 | [NM_003467.2](http://www.ncbi.nlm.nih.gov/entrez/viewer.fcgi?val=NM_003467.2) | Hs00237052_m1** |  |
| CXCR6 | chemokine (C-X-C motif) receptor 6 | NM_006564.1 | 5´-gcaaagcatctctgctggt-3´  5´-gaacccatagtcttcatggtaatca-3´ | **#53** |
| CXCR7 | chemokine (C-X-C motif) receptor 7 | [NM_020311.2](https://qpcr.probefinder.com/showsequence.jsp;jsessionid=B049628F43C2CD28540E5844C011ABAF.worker5?seqNo=364988595) | 5´-cagttgttgcaaagtgctcag-3´  5´-cgggcaatcaaatgacct-3´ | **#36** |
| IL2 | interleukin 2 | NM_000586 | Hs00174114_m1** |  |
| IL2RA | interleukin 2 receptor, alpha | NM_000417.2 | **Hs00907779_m1**** |  |
| IL2RB | interleukin 2 receptor, beta | [NM_000878.2](http://qpcr.probefinder.com/showsequence.jsp;jsessionid=7D3FD090644AB74FB595F87A52F3EEC3?seqNo=2008915113) | **5´-**ttgaaagacacctggagttcg**-3´**  **5´-**cctgcttctgcttgagagtca**-3´** | **#52** |
| IL15RA | interleukin 15 receptor, alpha | [NM_001243539.1](https://qpcr.probefinder.com/showsequence.jsp;jsessionid=B049628F43C2CD28540E5844C011ABAF.worker5?seqNo=1362813671) | **5´-**acaacccccagtctcaaatg**-3´**  **5´-**tgccgtcgttactgtggag**-3´** | **#37** |
| IFNG | interferon gamma | NM_000619.2 | Hs99999041_m1** |  |
| T-bet | T-box 21, T-cell-specific T-box transcription factor | **NM_013351.1** | Hs00894392_m1** |  |
| PSMB2 | proteasome (prosome, macropain) subunit, beta type, 2 | NM_002794.3 | 5´-agagggcagtggaactcctt-3´  5´-aggttggcagattcaggatg-3´ | #50 |

**^¥^** Numbers of Locked Nucleic Acid (LNA) probes according to the commercially available library ([www.universalprobelibrary.com](http://www.universalprobelibrary.com)).

Primers were designed using ProbeFinder assay design tool (Roche Applied Science, Indianapolis, IN, USA)

* Gene sequences available online at <http://www.ncbi.nlm.nih.gov/>

**Primers/probe sets (Assays-on-Demand) were from Life Technologies Corporation, Carlsbad, CA, USA

**Table S3.** Binding sites between studied miRNAs and mRNAs for cytokines, chemokines, cytokine/chemokine receptors and transcription factor *T-bet* identified by the mirSystem. Each hatched field corresponds to a condition, where a given mRNA is targeted by given miRNA, and this interaction was detected by at least one of the target gene prediction algorithms (including DIANA, miRanda, miRBridge, PicTar, PITA, rna22, and TargetScan) integrated in mirSystem.

|  | Let-7c | Let-7d | miR-21 | miR-24 | miR-25 | miR-92a | miR-125a | miR-126 | miR-133a | miR-146a | miR-148a | miR-150 | miR-155 | miR181a | miR-199a | miR-202 | miR-204 | miR-206 | miR-212 | miR-214 | miR-222 | miR-223 | miR-302c | miR-424 | miR-503 |
| --- | --- | --- | --- | --- | --- | --- | --- | --- | --- | --- | --- | --- | --- | --- | --- | --- | --- | --- | --- | --- | --- | --- | --- | --- | --- |
| Chemokines and molecules with chemotactic properties | | | | | | | | | | | | | | | | | | | | | | | | | |
| CCL2 |  |  |  |  |  |  |  |  |  |  |  |  |  |  |  |  |  |  |  |  |  |  |  |  |  |
| CCL3 |  |  |  |  |  |  |  |  |  |  |  |  |  |  |  |  |  |  |  |  |  |  |  |  |  |
| CCL4 |  |  |  |  |  |  |  |  |  |  |  |  |  |  |  |  |  |  |  |  |  |  |  |  |  |
| CCL5 |  |  |  |  |  |  |  |  |  |  |  |  |  |  |  |  |  |  |  |  |  |  |  |  |  |
| CCL7 |  |  |  |  |  |  |  |  |  |  |  |  |  |  |  |  |  |  |  |  |  |  |  |  |  |
| CCL8 |  |  |  |  |  |  |  |  |  |  |  |  |  |  |  |  |  |  |  |  |  |  |  |  |  |
| CCL13 |  |  |  |  |  |  |  |  |  |  |  |  |  |  |  |  |  |  |  |  |  |  |  |  |  |
| CCL19 |  |  |  |  |  |  |  |  |  |  |  |  |  |  |  |  |  |  |  |  |  |  |  |  |  |
| CXCL2 |  |  |  |  |  |  |  |  |  |  |  |  |  |  |  |  |  |  |  |  |  |  |  |  |  |
| CXCL3 |  |  |  |  |  |  |  |  |  |  |  |  |  |  |  |  |  |  |  |  |  |  |  |  |  |
| CXCL9 |  |  |  |  |  |  |  |  |  |  |  |  |  |  |  |  |  |  |  |  |  |  |  |  |  |
| CXCL10 |  |  |  |  |  |  |  |  |  |  |  |  |  |  |  |  |  |  |  |  |  |  |  |  |  |
| CXCL11 |  |  |  |  |  |  |  |  |  |  |  |  |  |  |  |  |  |  |  |  |  |  |  |  |  |
| CXCL12 |  |  |  |  |  |  |  |  |  |  |  |  |  |  |  |  |  |  |  |  |  |  |  |  |  |
| CXCL16 |  |  |  |  |  |  |  |  |  |  |  |  |  |  |  |  |  |  |  |  |  |  |  |  |  |
| Chemokine and cytokine receptors | | | | | | | | | | | | | | | | | | | | | | | | | |
| CCR1 |  |  |  |  |  |  |  |  |  |  |  |  |  |  |  |  |  |  |  |  |  |  |  |  |  |
| CCR2 |  |  |  |  |  |  |  |  |  |  |  |  |  |  |  |  |  |  |  |  |  |  |  |  |  |
| CCR5 |  |  |  |  |  |  |  |  |  |  |  |  |  |  |  |  |  |  |  |  |  |  |  |  |  |
| CXCR3 |  |  |  |  |  |  |  |  |  |  |  |  |  |  |  |  |  |  |  |  |  |  |  |  |  |
| CXCR4 |  |  |  |  |  |  |  |  |  |  |  |  |  |  |  |  |  |  |  |  |  |  |  |  |  |
| CXCR6 |  |  |  |  |  |  |  |  |  |  |  |  |  |  |  |  |  |  |  |  |  |  |  |  |  |
| CXCR7 |  |  |  |  |  |  |  |  |  |  |  |  |  |  |  |  |  |  |  |  |  |  |  |  |  |
| Cytokines and transcription factor | | | | | | | | | | | | | | | | | | | | | | | | | |
| IFNG |  |  |  |  |  |  |  |  |  |  |  |  |  |  |  |  |  |  |  |  |  |  |  |  |  |
| IL2 |  |  |  |  |  |  |  |  |  |  |  |  |  |  |  |  |  |  |  |  |  |  |  |  |  |
| IL2RA |  |  |  |  |  |  |  |  |  |  |  |  |  |  |  |  |  |  |  |  |  |  |  |  |  |
| IL2RB |  |  |  |  |  |  |  |  |  |  |  |  |  |  |  |  |  |  |  |  |  |  |  |  |  |
| IL15RA |  |  |  |  |  |  |  |  |  |  |  |  |  |  |  |  |  |  |  |  |  |  |  |  |  |
| T-bet |  |  |  |  |  |  |  |  |  |  |  |  |  |  |  |  |  |  |  |  |  |  |  |  |  |

**Fig. S1**. The cytokine-cytokine receptor interaction pathway targeted by 25 candidate miRNAs. Molecules targeted by one single miRNA are yellow-colored, molecules targeted by two or more miRNAs are orange-colored. The schema of cytokine-cytokine interaction pathway (provided by Kyoto Encyclopedia of Genes and Genomes, KEGG) was generated by **DIANA-miRPath v3.0.**


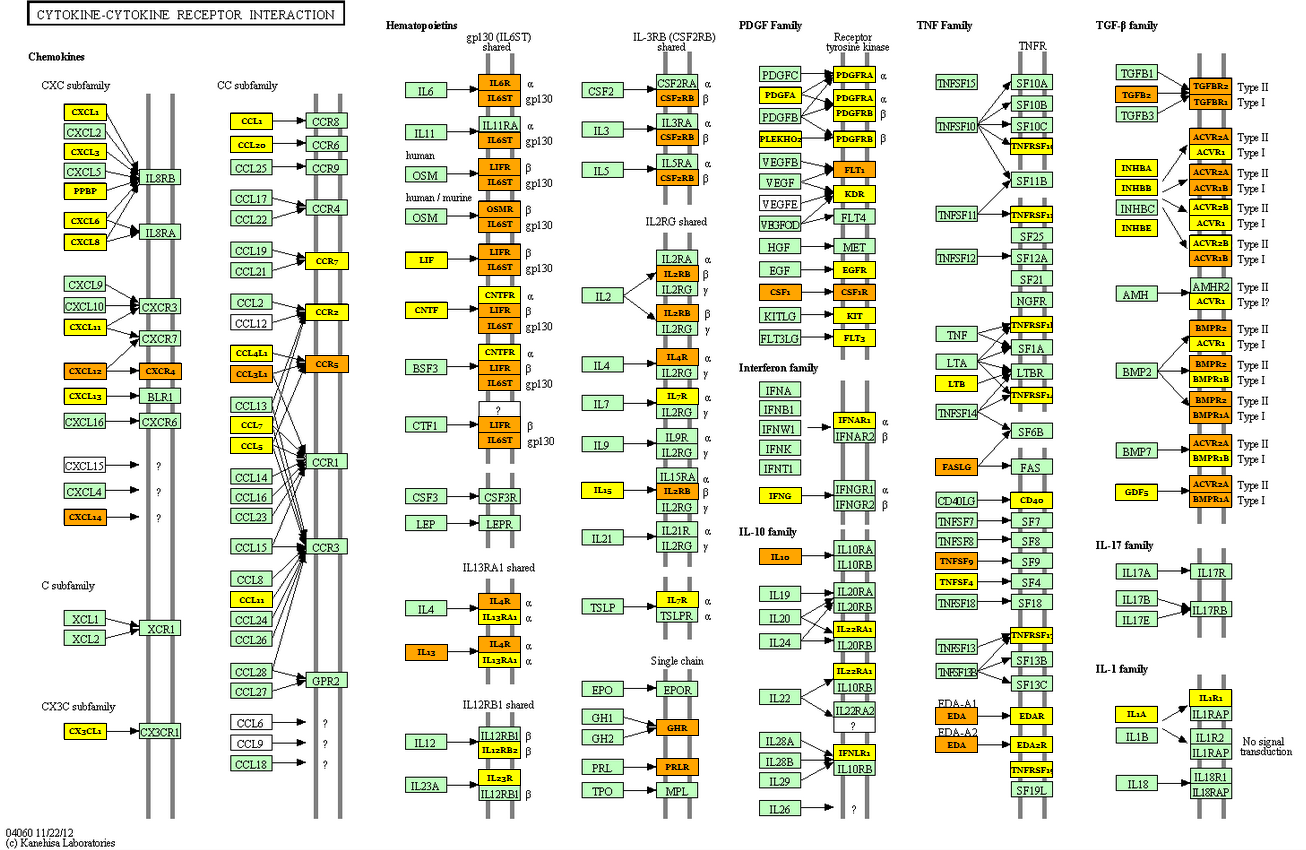


**Fig. S2.** A hierarchical agglomerative clustering analysis presented using heat maps for subgroups of patients with **A**) progressing and **B**) regressing sarcoidosis. The colour of each cell of the heat map corresponds to value of Spearman correlation coefficient between given miRNA-mRNA pairs. Legend: % ly, % of lymphocytes; abs ly, absolute number of lymphocytes/1ml BAL fluid.


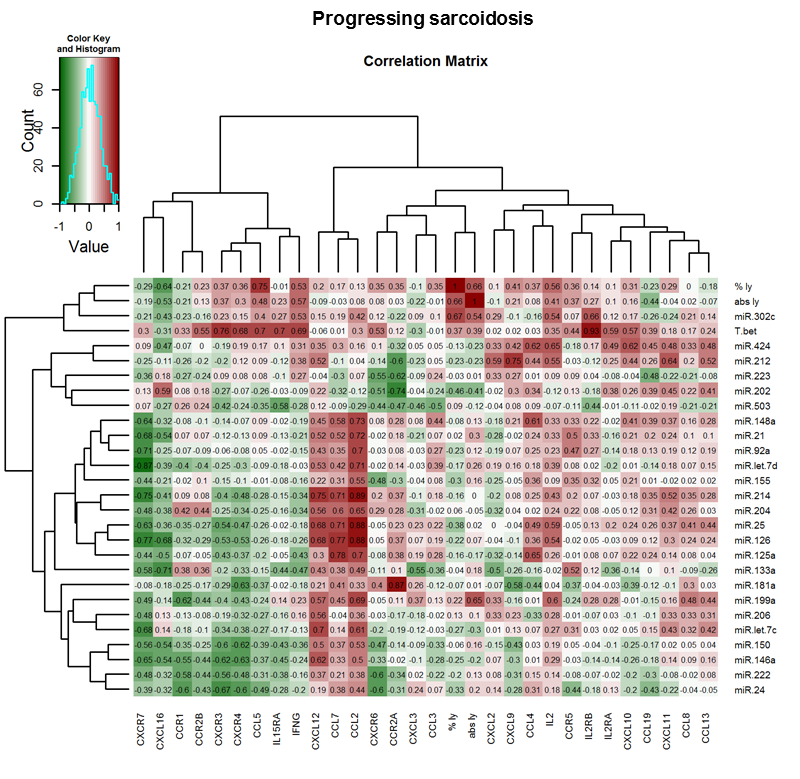


**A)**

**B)**


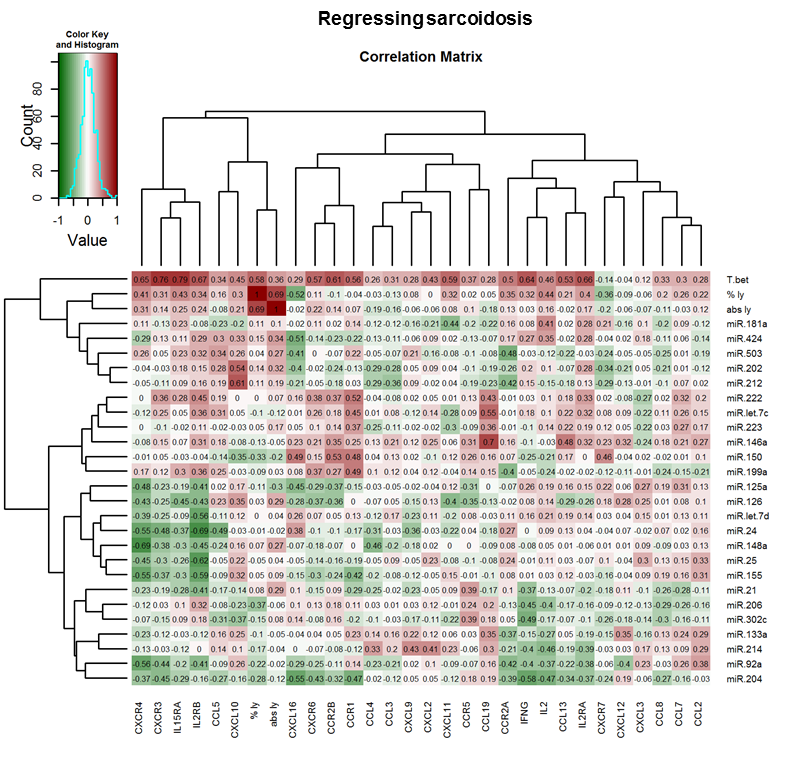

Supplement: Supplementary file 1 — Correlation network analysis reveals relationships between microRNAs, transcription factor T-bet and deregulated cytokine/chemokine-receptor network in pulmonary sarcoidosis [file 121378.f1.zip › 121378.f1.docx]
